# A bibliometric analysis of acupuncture for neurodevelopmental disorders: A Call for increased output and future research priorities

**DOI:** 10.1016/j.heliyon.2023.e22799

**Published:** 2023-11-23

**Authors:** Juexuan Chen, Huanjie Li, Dayuan Zhong, Fangwei Xu, Lu Ding, Chunzhi Tang, Chunguang Guan, Liming Lu, Jian Deng

**Affiliations:** a Department of Pediatrics of Traditional Chinese Medicine, Guangzhou Women and Children's Medical Center, Guangzhou, China; b Foshan Hospital of Traditional Chinese Medicine, Foshan, China; c Guangdong Provincial Hospital of Integrated Traditional Chinese and Western Medicine, Foshan, China; d Clinical Research Center, South China Research Center for Acupuncture and Moxibustion, Medical College of Acupuncture-Moxibustion and Rehabilitation, Guangzhou University of Chinese Medicine, Guangzhou, China; e Dongguan Eastern Central Hospital, Dongguan, China

**Keywords:** Acupuncture, Neurodevelopmental disorders, Bibliometric analysis, Mechanism, Review

## Abstract

**Objective:**

To perform a bibliometric analysis of published research on acupuncture for neurodevelopmental disorders and to provide new insights for future studies.

**Methods:**

Web of Science Core Collection was used to search for articles on acupuncture for neurodevelopmental disorders in children, from inception to Dec 4, 2022. VOSviewer and CiteSpace software were used for bibliometric analyses. VOSviewer was used to analyze and visualize the knowledge maps of the articles’ countries, authors, and institutions of origin, the journals and keywords. CiteSpace was used to visualize the dual-map overlay of the journals in which the articles were published and those publishing the articles they cited.

**Results:**

A total of 119 papers were retrieved. The highest number of publications came from China, followed by the United States and South Korea. The most frequently cited article was from the United States, followed by China. The most publications were from KyungHee University, followed by Sichuan University. Author Cho, Seung-hun from KyungHee University published the most articles. The Journal of Alternative and Complementary Medicine and Medicine published the most articles. The top three most frequently used keywords were “acupuncture”, “children”, and “complementary”.

**Conclusion:**

Research intensity and recognition, as well as collaboration within the field of acupuncture for treating neurodevelopmental disorders in children has increased. Research is generally diverse and comprehensive, and the neuro–endocrine–immune mechanism should be a new direction for further development. More basic research is also needed, to elucidate the therapeutic mechanisms, to standardize and validate the use of acupuncture for neurodevelopmental disorders.

## 1 Introduction

Neurodevelopmental disorders (NDDs) arise from changes in early brain development, resulting in behavioral and cognitive alterations in sensory and motor systems, speech, and language [1]. NDDs lead to a series of health problems in our society, affecting >3 % of children worldwide [2]. Children with NDDs have a higher prevalence of sleep disturbances [3] and their families experience significant psychological burdens, which have been particularly problematic during prolonged home isolation during the COVID-19 pandemic [4]. NDDs are a major public health concern, as they have a heterogeneous etiology and lead to impaired cognition, communication, adaptive behavior, and psychomotor skills. NDDs include intellectual disorder, autism spectrum disorder (ASD), attention deficit hyperactivity disorder (ADHD), tic disorder (TD), speech or language disorder, learning disorder, motor coordination disorder, stereotyped movement disorder, and other specific types. Important, typical characteristics of NDDs that lead to their grouping include childhood manifestation and early-onset neurocognitive abnormalities. In addition, these diseases often have multiple etiologies, which gives us reason to consider them together [1]. Many studies have suggested that shared molecular pathways could account for the multiple clinical symptoms of NDDs [2]. Accordingly, comorbidity of two or more of these disorders is frequently observed. For instance, a comorbid ADHD, TD, and learning disorder is common. Genetic studies indicate that different NDDs are associated with a channelopathy with similar underlying transcriptional mechanisms [5]. Thus, understanding the shared pathogenic mechanisms among NDDs may help explain their comorbidity and aid development of more effective treatments.

NDD treatment options are currently limited, largely because of their heterogeneous etiology [6]. Currently, only symptomatic treatments are available to manage the behavior problems common among patients with NDDs [7]. The available evidence-based treatment options for NDDs mainly aim to reduce basic behavioral symptoms [8]. The outlook for autism is even more concerning as there are currently no United States (USA) Food and Drug Association‐approved medications to address the core symptoms of this disorder [9].

Acupuncture is an important part of traditional Chinese medicine that has been systematically used for over 2000 years and is now rapidly gaining popularity in the West as an alternative and complementary practice for its clear therapeutic effects for nervous system diseases. In recent decades, network biology has emerged as a novel way to explain acupuncture's mechanisms of action [10]. A large volume of experimental acupuncture studies have shown that acupuncture's effects are related to participation among the nervous, endocrine, and immune systems and it activates the neuro–endocrine–immune (NEI) system through the body's large meridian network [11]. The NEI system further outputs the effect information to target organs through multilevel and multiple systems, finally acting on the disease network. Novel studies have addressed the disease–protein–gene network, showing that by calculating optimal parameters, acupuncture can change certain target genes by reverse derivation [12]. Similarly, through analysis of the acupuncture-target gene network, the diseases in which target genes are mainly involved can be calculated to identify those for which acupuncture has potential therapeutic effects [13]. Acupuncture's basic network biological mode may explain its therapeutic effects on a variety of NDDs. Specifically, use of acupuncture in ADHD [14], TD [15], and ASD [16] have undergone ongoing improvement and research exploration. However, little attention has been paid to comprehensively assessing the overall context and research priorities in the use of acupuncture to treat pediatric NDDs.

Bibliometrics was first introduced in the early 1900s. It became an independent discipline in 1969, after which it was widely applied in literature analyses. An interdisciplinary field, bibliometrics applies mathematical and statistical methods to quantify all knowledge carriers and identify the situation and frontiers in a field of study. Co-occurrence analysis of keywords, co-citation analysis, and burst of keywords can reflect global research trends and hotspots. Additionally, future directions of a research topic can be characterized and predicted by comparing the contributions of different countries, institutions, authors, and journals. Bibliometric analyses has already been applied across many acupuncture-related medical research fields, including fibromyalgia, insomnia, and magnetic resonance imaging.

Herein, CiteSpace [17] and VOSviewer [18] were used to analyze the research situation, hotspots, and trends concerning acupuncture for NDDs, with knowledge support.

## 2 Methods

### 2.1 Data sources and search strategies

To ensure complete coverage and authority of the data analyzed, Web of Science Core Collection was selected as the data source for searching articles on acupuncture for NDDs in children. The selected indexes were SSCI, SCI-Expended, and ESCI. The retrieval date was Dec 4, 2022, and the retrieved articles were published from the date of index inception to the retrieval date. The search strategy was as follows: topic= (acupuncture OR “acupuncture point*” OR electroacupuncture) AND topic= (“neurodevelopmental disorder*” OR “attention deficit hyperactivity disorder*” OR “attention deficit disorder with hyperactivity” OR “autism spectrum disorder” OR autistic disorder OR “Tourette syndrome” OR “tic disorder*” OR “intellectual disability*” OR “communication disorder*” OR “speech or language disorder*” OR learning disorder* OR “motor coordination disorder” OR “motor skills disorder*” OR “stereotyped movement disorder*”). We selected the key words related to neurodevelopmental disorder according to the categories in International Classification of Diseases 11th Revision (ICD-11) and Diagnostic and Statistical Manual of Mental Disorders, Fifth Edition (DSM-V). No restrictions were imposed on language or article type. Because data directly obtained through the search formula may have had problems like duplication or subject inconsistency, they were preprocessed to improve analysis quality. Two researchers independently screened each article title, abstract, and full text and decided on valid documents to include in final analyses. Disagreements were resolved through discussion.

### 2.2 Statistical analysis

Excel (version 2017; Microsoft) was used to manage article data and to create the publication dates figure. VOSviewer (version 1.6.18) and CiteSpace (version 5.8. R3) were used for statistical computing and graphics. VOSviewer was developed for constructing and viewing bibliometric maps [18]. Herein, we used this software to analyze collaborations and time trends among countries, institutions, and authors, the journals in which the articles were published, and to visualize knowledge maps. In the knowledge maps, node sizes represent the numbers of countries, citations, keywords, references, authors, and institutions, while line thickness represents the strength of the link. CiteSpace was used to construct a dual-map overlay of journals and to explore keywords with strong emergence.

## 3 Results

**Fig. 1:**
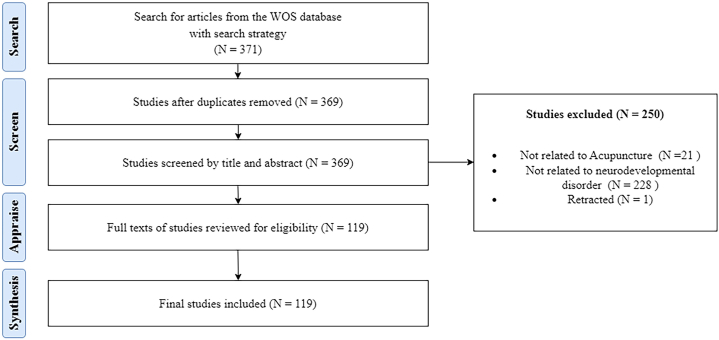
Screening flowchart.

Search outcomes identified 119 papers published from 1995 to 2022 (Fig. 1). The included articles were written by 545 authors from 194 organizations in 29 countries, published in 75 journals, and cited 5032 references in 1983 journals.

### 3.1 Annual trends in publication numbers

**Fig. 2:**
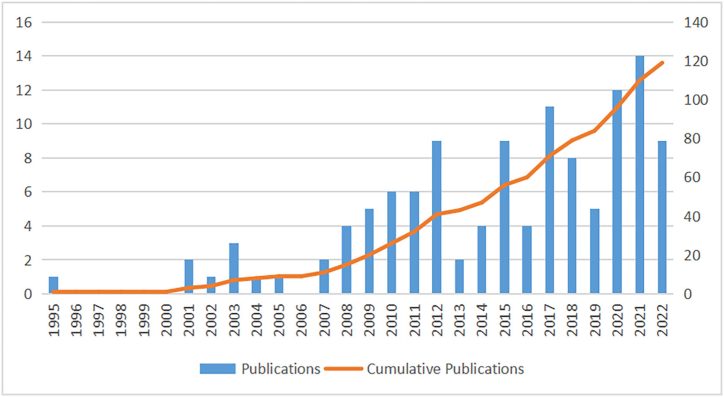
Number of annual publications on acupuncture for NDDs in children indexed on Web of Science Core Collection Database.

Changes in numbers of annual publications reflect the rate of the field's development and advancement, and the level of attention it received [19]. The number of articles published shows an increasing trend, with an annual average of fewer than two articles before 2007, significant increases after 2007, a reduction and low point during 2013 and 2014, and then increasing to a new height during 2020 and 2021 (Fig. 2).

### 3.2 Articles’ countries of origin

**Fig. 3:**
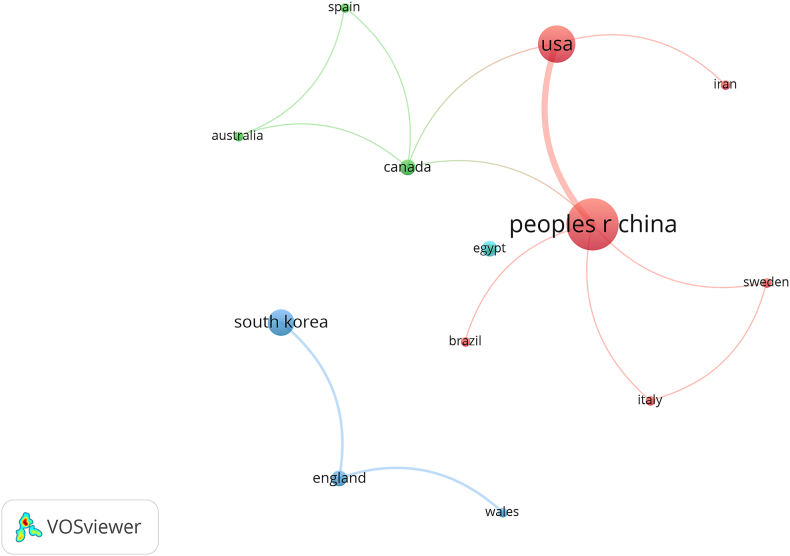
International cooperation network map.

Identifying publications based on their national origins provides insight into both the significance given to the subject within the country and the country's degree of influence over the subject. Overall, the 119 papers were published by 29 countries. For better visualization, we included only the 18 countries from which more than two articles were published (Fig. 3). International collaboration networks were divided into several clusters, while some countries did not participate in any international collaborations. Node size indicates the number of publications and line thickness indicates collaboration intensity. That international collaboration appears low across cooperative network clusters suggests that countries could strengthen their cooperation to support the growth of the discipline. As shown in Table 1, China had the highest number of publications (n = 47), followed by the USA (n = 25) and South Korea (n = 13). The country with the most frequently cited published articles was the USA (cited 490 times), followed by China (435 times) and Canada (161 times). Canada was ranked highest in terms of the average citations (32.20), and the USA second (19.60), demonstrating these countries' high publication quality. The relatively low average citations of articles published from England, China, and South Korea point to their needs for higher quality articles.

**Table 1:** Top six most productive countries.

Rank	Country	Publications	Citations	Average Citations
1	China	47	435	9.26
2	USA	25	490	19.60
3	South Korea	13	101	7.77
4	Canada	5	161	32.2
5	England	5	79	15.8
6	Germany	5	5	1

### 3.3 Articles’ institutions of origin

**Fig. 4:**
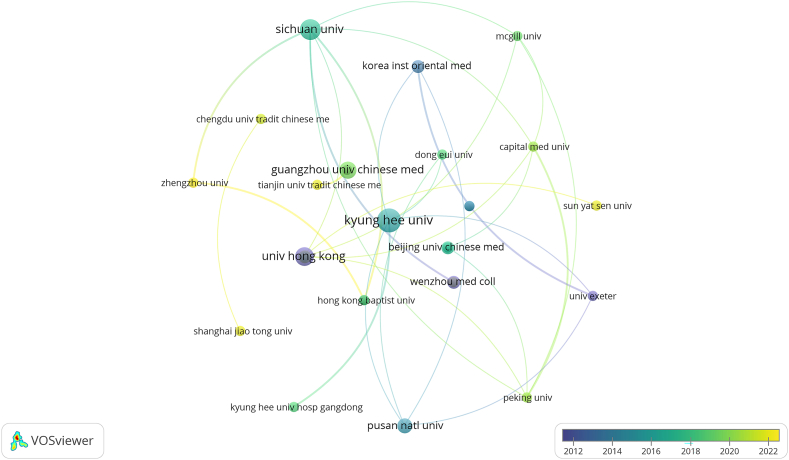
Map of institutions active on acupuncture for NDDs.

The institutional affiliations with the most publications are listed in Table 2. Kyung Hee University had the most (n = 9), followed by Sichuan University and the University of Hong Kong. In terms of average citations, the University of Hong Kong was highest (23.17), followed by Sichuan University (17.00) and the Wenzhou Medicine College (n = 14). The institutional collaboration co-occurrence map shows weak collaborative relationships among institutions, suggesting the need for greater collaboration (Fig. 4). China accounted for more than 60 % of the top nine most productive institutions, and Korea accounted for about 30 %, likely due to the high rates of acupuncture use in these countries. According to the timeline of articles published by institutions, Sun Yat Sen University, Zhengzhou University, Shanghai Jiao Tong University, and Tianjin University of Traditional Chinese Medicine contributed the most on this topic during 2022.

**Table 2:** Top nine most productive affiliations.

Rank	Affiliations	Country	Publications	Citations	Average Citations
1	KyungHee univ	Korea	9	77	8.56
2	Sichuan univ	China	7	119	17.00
3	Univ hong kong	China	6	139	23.17
4	Guangzhou univ Chinese med	China	5	14	2.80
5	Pusan natl univ	Korea	4	36	9.00
6	Korea inst oriental med	Korea	3	28	9.33
7	Wenzhou med coll	China	3	42	14.00
8	Huazhong univ sci & technol	China	3	19	6.33
9	Beijing univ Chinese med	China	3	21	7.00

### 3.4 Articles’ authors

**Fig. 5:**
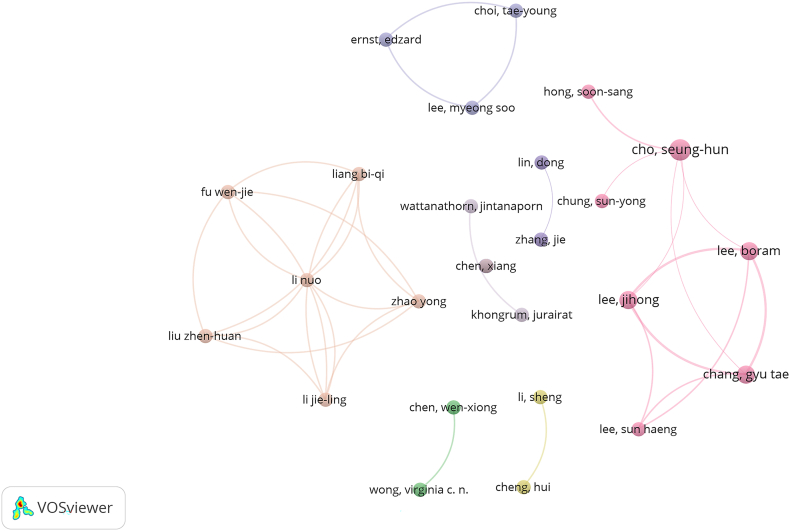
Map of authors' collaborations related to acupuncture for NDDs.

A total of 545 investigators made author-level contributions to the field, among which we selected 30 authors who had more than two papers for visual analysis (Fig. 5). The four most frequently published authors, with 13 papers each, accounted for 10.92 % of the total number of papers (Table 3). Author Cho, Seung-hun from KyungHee University published the most, followed by Chang, Gyu-tae and Lee, Boram, both also from KyungHee University. As depicted in Table 3, the four authors with the highest numbers of publications had similar average citations; they were also all from Korea, and their co-occurrence network was also explored.

**Table 3:** Top four most active authors.

Rank	Author	Country	Publications	Citations	Average Citations
1	Cho, seung-hun	Korea	4	23	5.75
2	Chang, Gyu tae	Korea	3	17	5.67
3	Lee, Boram	Korea	3	17	5.67
4	Lee, Jihong	Korea	3	17	5.67

**Fig. 6:**
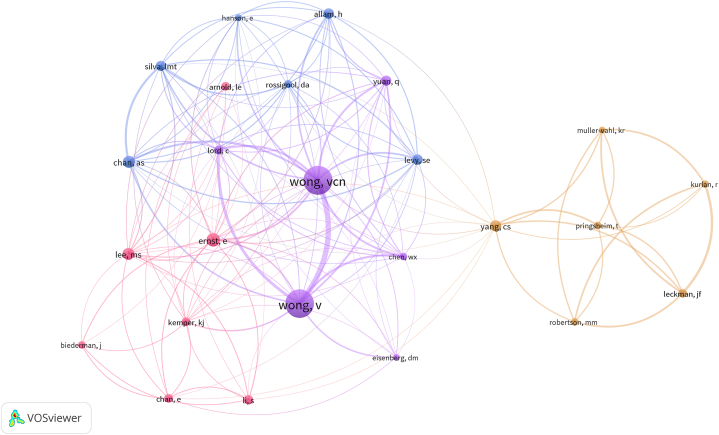
Map of co-cited authors related to acupuncture for NDDs.

The map of co-cited authors is in Fig. 6. For better visualization, we only included the 26 authors who were cited at least 10 times. The top five co-cited authors in frequency and centrality are listed in Table 4. Wong, VCN ranked first, followed by Wong, V, Ernst, E, Chan, AS, and Lee, MS. Among them, Lee, MS had the highest centrality.

**Table 4:** Top five most actively cited authors.

Rank	Author	Country	Frequency	Centrality
1	Wong, vcn	China (Hong Kong)	58	0.15
2	Wong, V	China (Hong Kong)	57	0.08
3	Ernst,e	England	23	0.00
4	Chan, as	China (Hong Kong)	20	0.06
5	Lee, ms	South Korea	20	0.37

### 3.5 Journals and cited-journals analysis

**Fig. 7:**
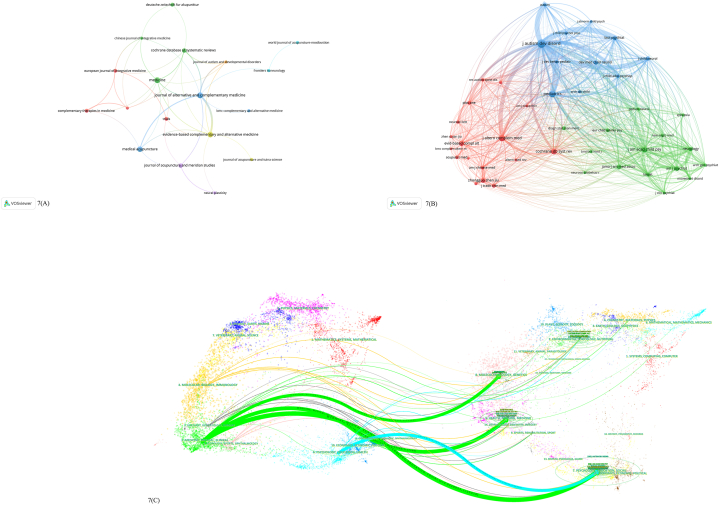
A. Map of journals publishing acupuncture for NDDs. B. Map of cited journals publishing acupuncture for NDDs. C. Dual-map overlay of A and B (publishing journal on left; cited journal on right; line path represents the citation relationship).

VOSviewer was used to count the journals that published the included articles (Fig. 7A and B). The 119 total articles were published by 75 academic journals, all with highly-ranked reputations. As shown in Table 5, the top ranked journals in terms of number of published included articles were the Journal of Alternative and Complementary Medicine (7 publications, impact factor [IF]: 2.381, journal citation report [JCR]: Q1), Medicine (7 publications, IF: 1.817, JCR: Q2), and Complementary and Alternative Medicine (6 publications, IF: 2.650, JCR: Q2). An examination of the journals cited within the included articles reveals those journals' contributions to the field's knowledge base (Table 5). Of the 1983 cited journals, three were cited more than 100 times: Journal of Autism and Developmental Disorders (IF: 4.345, JCR: Q1), Pediatrics (IF: 9.703, JCR: Q1), and Journal of Alternative and Complementary Medicine (IF: 2.381, JCR: Q3).

**Table 5:** Top 10 journals and co-cited journals related to acupuncture for NDDs.

Rank	Journals	Publications	IF and JCR division (2022)	H-index	Cited journals	Ciation	IF and JCR division (2022)	H-index
1	Journal of alternative and complementary medicine	7	2.381/Q1	80	JOURNAL OF AUTISM AND DEVELOPMENTAL DISORDERS	185	4.345/Q1	NA
2	Medicine	7	1.817/Q2	135	PEDIATRICS	114	9.703/Q1	311
3	Evidence-based complementary and alternative medicine	6	2.650/Q2	72	JOURNAL OF ALTERNATIVE AND COMPLEMENTARY MEDICINE	102	2.381/Q1	80
4	Medical Acupuncture	6	N.A./Q3	N.A.	Cochrane Database of Systematic Reviews	87	12.008/Q1	244
5	Cochrane database of systematic reviews	4	12.008/Q1	244	JOURNAL OF THE AMERICAN ACADEMY OF CHILD AND ADOLESCENT PSYCHIATRY	86	13.113/Q1	222
6	Journal of acupuncture and meridian studies	4	N.A./Q2	N.A.	Evidence-based Complementary and Alternative Medicine	73	2.650/Q2	72
7	European journal of integrative medicine	3	1.813/Q2	18	Zhongguo Zhen Jiu	69	NA	NA
8	Trials	3	2.728/Q2	64	AMERICAN JOURNAL OF PSYCHIATRY	62	19.242/Q1	325
9	Complementary therapies in medicine	3	3.335/Q1	55	JAMA-JOURNAL OF THE AMERICAN MEDICAL ASSOCIATION	58	157.335/Q1	622
10	deutsche zeitschrift fur akupunktur	3	N.A.	N.A.	DEVELOPMENTAL MEDICINE AND CHILD NEUROLOGY	53	4.864/Q1	129

The dual-map overlay (Fig. 7C) shows that most of the journals in which the included articles were published subsume three subject areas: medicine/medical/clinical, molecular/biology/immunology, and neurology/sports/ophthalmology. Most of the journals cited within the included articles were within the fields of molecular/biology/genetics, health/nursing/medicine, and psychology/education/social. The lines in the figure represent the links between published articles and their cited references. The psychology/education/social and health/nursing/medicine fields were cited most frequently by publications in other fields, indicating their importance to this knowledge base.

### 3.6 Articles’ keywords

**Fig. 8:**
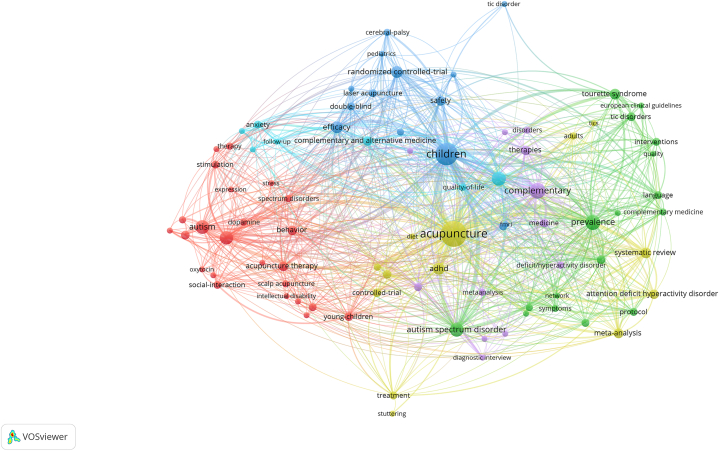
Map of co-occurring keywords related to acupuncture for NDDs.

Keywords, especially those used with high-frequency, usually represent research subject hotspots. Keywords analysis, keyword clusters, and burst detection are among the outcomes. We collected 601 keywords from the 119 included articles. The three keywords with the highest frequency of occurrence were “acupuncture” (n = 52), “children” (n = 39), and “complementary” (n = 20). Excluding those keywords related to therapy and population, the four keywords with the highest frequency were “prevalence” (n = 18), “alternative medicine” (n = 16), “autism spectrum disorder” (n = 15) and “autism” (n = 15) (Fig. 8). These results indicate that these articles focused mainly on epidemiology, diseases, and non-pharmacological research.

**Fig. 9:**
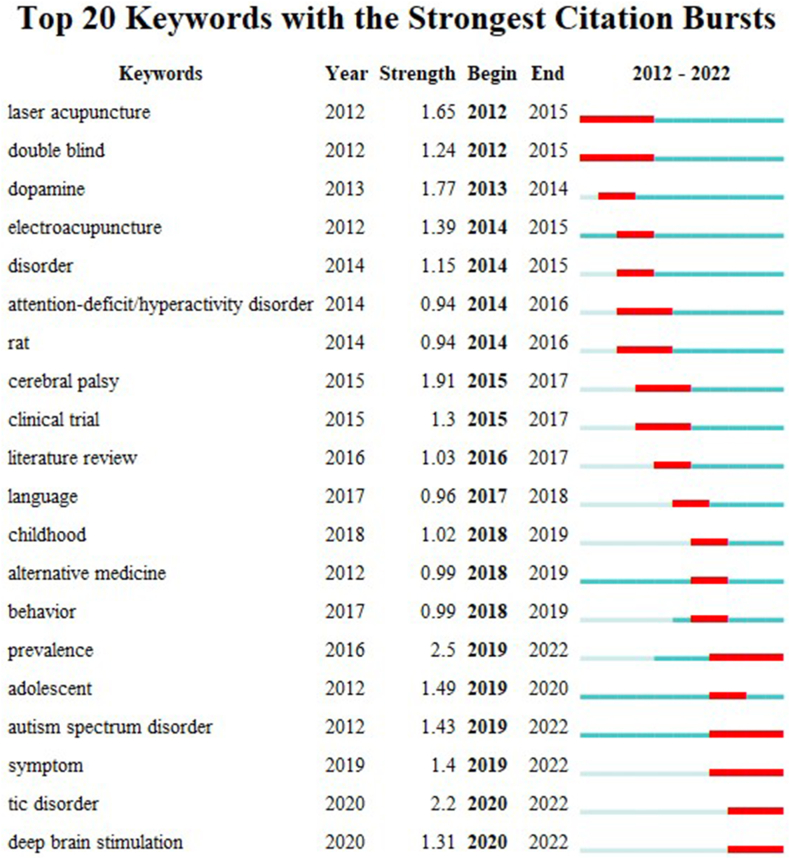
Top 20 keywords with the strongest citation bursts. Strength values indicate citation frequency. Red bars indicate time period during which the keyword appeared.

The burst detection results are shown in Fig. 9, indicating the field's research frontiers at specific times. The strength values indicate citation frequency and the red bars indicate the time period in which the keyword appeared. The high-strength terms were “prevalence”, “cerebral palsy”, “dopamine”, “laser acupuncture”, and “adolescent”.

## 4 Discussion

### 4.1 Study of acupuncture for neurodevelopmental disorders needs to be further diversified and international

To our knowledge, this is the first bibliometric analysis of acupuncture for NDDs. Acupuncture has unique advantages [20] and important roles in the prevention and treatment of NDDs, as it has been shown to effectively alleviate symptoms, improve behavior, and delay progression [21]. Herein, CiteSpace and VOSviewer were used to conduct a bibliometric study of acupuncture research on NDDs from 1995 to 2022. We compiled general data regarding publications of studies on acupuncture for NDDs, research hotspots, and research trends. The change in annual publication trends indicates dynamic changes from 1995 to 2022. Although the overall number of publications increased, growth in the number of annual publications was inconsistent.

The first research report on acupuncture for NDDs treatment was published in 1995. The highest number of publications was in 2021. As a quintessence of Chinese wisdom, acupuncture is a key component of traditional Chinese medicine, and has made significant contributions to China's healthcare system. Acupuncture has also gained popularity among researchers outside China after the initial reports of its beneficial effects on neurodevelopmental disorders such as tics and ADHD. For example, researchers located in the USA, Korea, Canada, England, Germany, Australia and Brazil have also conducted studies on the effect of acupuncture in treating NDDs. In addition, acupuncture therapy was mentioned in the Singapore's ADHD clinical practice guidelines [22].

Institutions that published research on acupuncture treatment for NDDs were mainly oriental medicine and comprehensive universities in China and Korea. However, our analysis showed that the research carried out in these institutions was not conducted during the same time periods. For example, The University of Hong Kong was the first to start the research, while the study of acupuncture for NDDs has been conducted by institutions in Korea since 2015. In addition, the geographic focus of research in this field gradually shifted to China after 2020. Although these institutions do not work in a broadly collaborative manner, collaborative efforts by multiple researchers and research institutions have increased. This may be related to the exchange programs at many universities, which offer a large number of researchers and students the opportunity to work and study abroad [23].

Analysis of keyword frequency in the included articles showed that studies of acupuncture for NDDs were primarily clinical research, systematic reviews, and epidemiology studies. Keyword co-occurrence analysis indicated that specific words were associated with different research types. For instance: the terms “acupuncture therapy”, “behavior”, and “safety” were associated with clinical research; “complementary and alternative medicine”, “meta-analysis”, and “randomized controlled trial” were related to systematic reviews; and epidemiology was often associated with the terms like “children” and “prevalence”.

We also sorted the included articles according to NDDs. Most of the articles addressed ASD, followed by ADHD, Tourette syndrome (or TD), speech fluency disorder, and intellectual disabilities. This illustrates the wide applicability of acupuncture treatment across NDDs. We also categorized the 119 articles according to study type. There were 48 systematic reviews or meta-analyses, 30 clinical studies, 11 surveys, 9 case reports, 13 protocols, and 7 experimental studies (Table 6). This indicates that previous research on acupuncture and NDDs is multifaceted, but need to be increased.

**Table 6:** Designs of included studies.

Study Design	Number of publications
System review or meta-analysis	48
Clinical study	30
survey	11
case report	9
protocol for systematic review	8
experimental study	7
protocol for clinical study	5
letter	1

### 4.2 Acupuncture advantages for attention deficit hyperactivity, autism spectrum, and tic disorders

In the analysis of keyword burst intensity, the disease-related keywords like “attention-deficit/hyperactivity disorder”, “autism spectrum disorder”, and “tic disorder” appeared frequently. This is related to the fact that the research on these NDDs have been field hotspots, especially ASD and TD, which have received a lot of attention in recent years, perhaps because of the significant increase in the use of acupuncture in pediatric care [24]. Analyses of the recent, included articles revealed that most explored the efficacy and safety of acupuncture as a complementary and alternative therapy for improving general symptoms of patients with NDDs. For example, Zhuo [25] described acupoint stimulations increased prefrontal cortex blood flow in children with ADHD, with mild adverse events. Hai et al. showed therapeutic effects of acupuncture for TD, especially vocal tic, in children [26]. Hong et al. found that patients who received acupuncture treatment without ADHD medications demonstrated significantly better cognitive function compared with the control group [27]. Many other researchers have focused on comparing different acupuncture therapies. For example, scalp acupuncture is used commonly to treat NDDs, due to its efficacy for improving verbal communication in both natal and regressive autism [28]. Ear, or auricular, acupuncture, which is a component of the body's micro-acupuncture system, significantly reduced ADHD symptoms in children [29]. In addition to traditional acupuncture, Japanese-style acupuncture [30,31] and Korean-style acupuncture [32,33] have also attracted research attention, emphasizing the diversity of acupuncture therapy.

To enhance the quality of research evidence, systematic reviews and meta-analysis are the most popular research types in the field of acupuncture for NDDs. One study systematically summarized the acupuncture therapy prescriptions for TD, showing that Bai-hui (DU20) and Feng-chi (GB20) are the most commonly used acupoints, and that the Governor Vessel has a significant role in TD treatment [34]. Another, similar review identified nine main channels that contribute to autism symptoms [35]. The increasing number of protocol-type articles shows that research designs in this field have become more rigorous and scientific, consistent with the rapid development of evidence-based medicine in recent years [[36], [37], [38], [39]].

### 4.3 Future research: neuro–endocrine–immune system mechanism of acupuncture in neurodevelopmental disorder treatment

The mechanisms of action of acupuncture for NDDs remain unclear [40], and are a prevalent issue mentioned in previous studies [[41], [42], [43]]. Consistent with this need, new research directions have emerged in the past five years. Studies on issues related to acupuncture and NDDs increasingly explore mechanisms. Most experimental studies included herein were published after 2015, when more findings and conclusions were from research on acupuncture for NEI system regulation.

Multiple studies have proposed autoimmune dysfunction in the pathogenetic mechanism of Tourette syndrome and related NDDs, including autism and ADHD [44,45]. Chronic inflammation and increased oxidative stress may lead to immune dysfunction, resulting in elevated TH2 induction and thereby increased Immunoglobulin E levels, which may cause ADHD [46]. These are similar to the potential mechanisms of ASD, including gastrointestinal tract microbiota and oxidative stress [47]. In addition, acupuncture can balance the release of stimulatory and inhibitory mediators in some cases [48]. It is generally understood that localized acupuncture-mediated anti-inflammatory effects involve regulation of multiple immune cell populations and their functions [49]. Acupuncture at specific acupoints regulates the NEI network [50,51].

Analysis of the included articles indicated that the NEI mechanism of acupuncture in treating NDDs is worthy of study [52]. One study suggested that autistic-like rats that received acupuncture showed a decreased malondialdehyde level in the cerebral cortex, which might explain its positive effect [53]. Similarly, another study showed that acupuncture on HT7 decreased oxidative stress and IL-6 expression, and improved GABAergic function [54]. Moreover, there is evidence that transcutaneous electrical acupoint is beneficial for neuron development in an autism-like animal model, and that activating the neurotrophic signaling pathway may be a mechanism of acupuncture treatment of ASD [55]. In addition to animal studies, several reviews have pointed out the value of acupuncture in neuroimmune network modulation in NDDs. Acupuncture may also promote social interaction by directly regulating the serotonergic or dopaminergic systems, or by facilitating the release of neurotransmitters in the brain's reward region via oxytocinergic systems [56]. The potential therapeutic effects of acupuncture-induced activation of brain-derived neurotrophic factor in the treatment of autism have also been demonstrated. However, further confirmation of these results is required, as is further investigation of potential differences between different NDDs through advances in developing animal models [57].

### 4.4 Study limitations

Some study limitations must be considered. First, we only included English literature available in the Web of Science database and did not examine other databases (e.g., PubMed, CNKI, WANFANG). Accordingly, we may have overlooked relevant published articles on the study subject. Second, we estimated the number of published studies reporting the use of acupuncture for NDDs but did not go into their details, such as the quality, design, sample size, intervention, outcome, etc. Third, we briefly discuss the related mechanisms reported in published studies but did not provide an in-depth analysis of these, due to the complexity and diversity of the molecular pathways involved in NDDs and acupuncture.

### 4.5 Conclusion

This study provided insights into the published research on acupuncture in the prevention and treatment of NDDs, and addressed reporting aspects like publication year, journal, country, institution, and keywords. Analysis of the current research status revealed that this field is at an early development stage and that China needs to strengthen collaborations with other countries. Hot topics and trends in acupuncture for NDDs mainly involve TD, ASD, and ADHD. In addition, the relevant literature was analyzed to identify that the NEI system is a potential mechanism of acupuncture. However, to date, comprehensive, in-depth research addressing the molecular mechanisms of acupuncture remain scarce. Further optimization experimental research is thus needed to identify therapeutic mechanisms and promote better uses of acupuncture in NDD treatment. This will be an important focus for current and future research in this field.

## Author contributions

Juexuan Chen and Huanjie Li contributed equally. JD and LML conceptualized the study design. JXC and HJL wrote the draft and complement the methods of this study. DYZ and FWX helped search and screen the titles and abstracts of articles. LD and CGG helped confirm the data. CZT and LML helped resolve differences. All authors approved the final version of the manuscript.

## Funding

No funding was received for this work.

## Ethical statement

No ethical approval was required as this study did not involve human participants or laboratory animals.

## Data availability

The data that support the findings of this study are available from the corresponding author upon reasonable request.

## Declaration of competing interest

The authors declare that they have no known competing financial interests or personal relationships that could have appeared to influence the work reported in this paper.

## Abbreviations

ADHD: attention deficit hyperactivity disorder
ASD: autism spectrum disorder
NDDs: neurodevelopmental disorders
NEI: neuro–endocrine–immune system
TD: tic disorder
